# Neuropsychiatric Aspects of Sotos Syndrome: Explorative Review Building Multidisciplinary Bridges in Clinical Practice

**DOI:** 10.3390/jcm13082204

**Published:** 2024-04-11

**Authors:** Sigita Lesinskiene, Reda Montvilaite, Kamile Pociute, Ausra Matuleviciene, Algirdas Utkus

**Affiliations:** 1 Clinic of Psychiatry, Institute of Clinical Medicine, Faculty of Medicine, Vilnius University, LT-03101 Vilnius, Lithuania; kamile.pociute@mf.vu.lt; 2 Faculty of Medicine, Vilnius University, LT-03101 Vilnius, Lithuania; reda.montvilaite@mf.stud.vu.lt; 3 Department of Human and Medical Genetics, Institute of Biomedical Sciences, Faculty of Medicine, Vilnius University, LT-03101 Vilnius, Lithuania; ausra.matuleviciene@mf.vu.lt (A.M.); algirdas.utkus@mf.vu.lt (A.U.)

**Keywords:** Sotos syndrome, mental health, child, adolescent, personalized treatment, autism spectrum disorder, behavioral phenotype, cognitive profile

## Abstract

**Background:** Sotos syndrome is a genetic disorder caused by *NSD1* gene (nuclear receptor binding SET domain containing protein 1) variants and characterized by overgrowth, macrocephaly, learning disabilities, and co-occurring neuropsychiatric symptoms. **Methods:** Literature sources published in 2002–2023 were selected and analyzed from PubMed and Google Scholar databases. **Results:** Neuropsychiatric symptoms are observed among children and adolescents with Sotos syndrome. The majority have intellectual disabilities or borderline intellect. Verbal IQ is higher than performance IQ. Individuals display difficulties in expressing language. Aggression is reported by parents. Children express autistic behavior, ADHD, anxiety based on phobias, and early bedtime-wake times. **Conclusions:** Sotos syndrome is associated with neuropsychiatric disorders in children. Slow intellectual and language development, aggressive outbursts, anxiety, autism spectrum disorder, and hyperactivity are present in the newest studies. Comprehensive assistance is needed for Sotos syndrome patients in responding to areas of difficulty. There is still a lack of research on the developmental characteristics of these children and the possibilities of improving psychosocial adaptation by providing multidisciplinary long-term medical, educational, and social care.

## 1. Introduction

Sotos syndrome is a rare genetic disorder with an incidence of 1 in 14,000 live births. The syndrome was first recognized in 1964 by a team led by Juan Sotos, who observed patients with similar clinical features. Pre- and postnatal overgrowth is characterized by macrocephaly (OFC ≥ 98th percentile) with characteristic facial features, advanced bone age, and intellectual disability [1]. Later, in 1994, the scientists Cole and Hughes listed these features as the four major diagnostic criteria of Sotos syndrome. Overgrowth was defined as excessive height, weight, and bone age inconsistent with the age of the person; signs of acromegaly include a prominent forehead, high frontal hairline, down-slanting palpebral fissures, and a protruding chin [2]. Other health observed issues include vascular and cardiac anomalies, genitourinary anomalies in children, neonatal jaundice, neonatal hypotonia, seizures, and scoliosis. A growing number of larger scale studies on such patients have associated these physical features as the main phenotype of Sotos syndrome [3].

Until 2002, the diagnosis of Sotos syndrome was based mainly on clinical features. However, recent data confirm that the disease also manifests itself at the molecular level. A genetic study for the first time identified variants in the epigenetic machinery genes attributed to Sotos syndrome in a Japanese population [4]. Specific microdeletion of the chromosomal region 5q35 flanking the *NSD1* gene was present in a large number of participants clinically diagnosed with the syndrome. Interestingly, individuals with this microdeletion have less pronounced rapid growth and more severe mental retardation [5]. In individuals of other ethnicities, the cause is intragenic variants in the *NSD1* gene, which occurs in about 90% of cases [6]. Testa et al. examined a cohort of 1530 patients with clinical suspicion of Sotos syndrome, identifying *NSD1* variants in 292 patients. A total of 269 patients were carriers of intragenic gene variations, 13 of 5q35 microdeletions of the entire *NSD1* gene, and 10 of exon gene deletions. A total of 119 patients were carriers of previously undescribed novel intragenic variants. Out of the 115 identified variants of uncertain significance (VUSs), 32 underwent reclassification. Among these, 25 missense *NSD1* VUSs (78.1%) were reclassified as likely pathogenic or likely benign, indicating a notably significant shift in class [7].

Detailed neuropsychiatric investigations as well as molecular studies of Sotos syndrome have begun only recently. Neuroimaging studies revealed that Sotos syndrome often involves minor neuroimaging abnormalities, such as trigeminal prominence in 90% of cases, occipital horn prominence in 75% of cases, and ventriculomegaly in 63% of cases. Enlargement of cerebral ventricles is frequently observed. This syndrome can also be associated with non-specific neurological abnormalities such as prominent cortical sulci, absent corpus callosum, cavum septum pellucidum, and cavum velum interposition [8]. Distinctive neuropsychiatric features associated with the disease are already observed in early childhood during neurodevelopmental assessment tests. During psychological investigation, a wide spectrum of behavioral, cognitive, and emotional changes are noted. Individuals diagnosed with Sotos syndrome exhibit varying levels of intellectual capabilities, ranging from complete independence to full dependence [9]. Most children’s intellectual abilities do not correspond to their age, and the difference between verbal and performance IQ is significant. Patients experience speech and language delays since early childhood and difficulties expressing language are reported [10]. A 2002 review of the literature on the neuropsychiatric aspects of Sotos syndrome described that patients with Sotos syndrome are at higher risk of developing intellectual and cognitive impairments of varying severity. These patients may also have impaired social adjustment, hyperactivity, phobias, temper tantrums, irritability, and feeding and sleeping disorders [11]. There is a lack of new studies summarizing the various neuropsychiatric aspects of Sotos syndrome. 

In this review, we aim to provide a comprehensive description of the neuropsychiatric characteristics of Sotos syndrome found in the literature and to emphasize the importance of organizing comprehensive multidisciplinary systematic help for these patients in clinical practice.

## 2. Materials and Methods

The literature review was conducted using the PubMed database. Publications were selected from 2002 to 2023 using the following keywords and their combinations: Sotos syndrome and psychiatric symptoms, psychiatric disorders, cognitive profile, behavioral phenotype, intellectual disability, autism spectrum disorder, sleep disorder, multidisciplinary help, or clinical practice. A total of 177 articles were found. Only publications written in English were included. Case reports were excluded. Ten articles that described neuropsychiatric aspects were found. Additionally, a search was conducted in the Google Scholar database. An additional article by Goulding-Talbot, J. was found. A total of 11 articles were included in the review (Table 1). Of the included studies, eight were cross-sectional, one was a systematic review, and two were literature reviews. We decided to include in our study the summarized information from the two literature reviews to increase conceptual clarity. The results were divided into eight sections: neurobehavioral phenotype, intelligence quotient, language, aggression and tantrums, autistic traits, attention deficit hyperactivity disorder, anxiety, and unusual sleep patterns.

## 3. Results

### 3.1. Neurobehavioral Phenotype in Sotos Syndrome

Despite the fact that Sotos syndrome was described in the 20th century, only in modern literature are the neuropsychiatric symptoms recognized as being syndrome specific. Like other Mendelian disorders of the epigenetic machinery (MDEM), Sotos syndrome is classified as a neurodevelopmental disorder, meaning the patient’s neuropsychiatric profile changes through life. The most frequent neurologic features for infants and toddlers are hypotonia and delayed acquisition of both fine and gross motor skills. As a child develops hypotonia, it usually becomes less prominent, like in other MDEMs. However, motor skill acquisition does not progress as promptly in early childhood as expected, according to developmental assessments.

Furthermore, hypotonia and delayed motor skills (especially fine motor) become less observed although they still persist. Cognition and behavioral difficulties start manifesting in older children with Sotos syndrome, thus leading to declining results in intellect tests and language assessments, and disrupted emotional state and psychiatric disorders, such as autism spectrum disorder or attention deficit hyperactivity disorder (ADHD) [12]. However, data including only clinical neuropsychiatric aspects with no presented relationship to molecular features present only superficial information about cognition in Sotos syndrome. Consequently, a detailed genetic assessment may become a crucial aspect in solidifying the neurocognitive phenotype in future studies.

### 3.2. Intelligence Quotient (IQ)

Intellectual disability is one of the most common signs associated with genetic syndromes, especially with MDEMs. Cognitive functioning is mainly affected and the intelligence quotient (IQ) is a helpful standardized indicator to measure it. Children prior to age 18 with suspected intellectual disability are assessed following the American Association on Intellectual and Developmental Disabilities guidelines. The IQ measurement includes the investigation of brain areas responsible for learning, problem solving, adaptive skill development, and independence [22].

A wide-ranging review by Lane at al. in 2016 summarized 34 studies that researched cognitive and/or behavioral aspects of Sotos syndrome. These studies put emphasis on the majority of individuals with Sotos syndrome having variable IQ, which ranges from mild intellectual disability (IQ = 50–69) to being in the borderline cognitive range (IQ = 70–84). However, there are some cases presenting average intellect or even severe intellectual disability [13]. 

In addition, in several reviewed studies that included full-scale IQ scores (FSIQ), performance and verbal IQ scores were reported. The results implied that Sotos patients perform better in verbal IQ (VIQ) than performance IQ (PIQ) tasks. Nevertheless, another article by Harris et al. in 2019 as well as a later article by Lane et al. (2019) suggested that the mentioned subdomain scores were incomplete since more specific cognitive domains were not investigated [12,14]. In general, MDEMs determine intellectual disability by ~19% and overgrowth combined with intellectual disability by ~45%, with Sotos syndrome being the leading cause according to the latest data [12]. However, other syndromes also cause the H3K36 molecule methylation that results in similarly disrupted neurologic development and skeletal growth. Thus, Sotos recognition becomes more complicated and epigenetic differential diagnosis should be included in completing the comprehensive cognitive Sotos profile.

The deeper cognitive reasoning of Sotos syndrome has been researched since 2019 and is based on examples of other neurodevelopmental disorders, such as Williams or Down syndrome. In Lane et al.’s most recent study, these criteria were evaluated using the third edition British Ability Scale (BAS3) to choose the population of adults and children with Sotos syndrome. To complete the cognitive profile, cluster score analysis by ANOVA was used. Patients demonstrated four different patterns that together constituted the cognitive profile of the syndrome. The received data of 52 participants supplemented the understanding of strengths and weaknesses of the intellectual aspect of Sotos. The results highlighted a stronger verbal ability and visuospatial memory and weaker non-verbal ability and quantitative reasoning [14]. The suggested findings of this study could be important in considering an adaptive education for children with Sotos syndrome.

### 3.3. Language

The assessment of speech and language abilities plays an integral part in evaluating a child’s development because impaired language may be a manifestation of a genetic disorder [23]. The primary language delay, which includes developmental speech and language delays, expressive language disorder, and receptive language disorder in Sotos syndrome, was reviewed in Lane et al.’s article in 2016 [13]. Although the study suggested examining language abilities in relation to general intellectual development, the results reliably defined this neuropsychiatric aspect of Sotos syndrome. According to Lane’s research and a previously performed cohort study by Sarimski in 2003, the language comprehension and expression of Sotos patients were insignificantly impaired in comparison with their general level of intellectual functioning and are consisted with FSIQ scores [13,15]. Lane et al.’s findings linked the delicate language impairment with the higher verbal IQ score and thus suggested that absolute communication deficits are not a characteristic feature of Sotos syndrome [13] 

However, several previous cases that were mentioned in Lane’s review reported different results. The investigated language abilities may have indicated that Sotos patients display primary language delays when compared to representatively developing groups. Individuals with Sotos especially experience difficulties with expressive rather than receptive language. Unfortunately, the level of intellectual capacity in Sotos syndrome participants was not reported while evaluating their language ability. This shortcoming in the assessments of older studies was also reviewed in the article by K. Sheth et al. in 2015 [16]. Also, in earlier studies, the results were not compared to control groups matched for intellectual functioning and were only based on clinical observation. Consequently, a significant language delay is not presented in Sotos syndrome individuals and specific communication impairments require additional investigation.

### 3.4. Aggression and Tantrums

The behavioral profile of Sotos syndrome was defined in several previous studies around the year of 2000. Increased aggression is believed to be one of the behavioral problems in children with Sotos diagnosis, although results of this particular aspect are debatable. As is discussed in Lane et al.’s review in 2016, the majority of this research was based on subjective parents’ opinion on children’s emotions and behavior. In addition, the chosen sample size was limited. This is why comparing younger Sotos children with typically developing individuals may have created misleading knowledge about the occurrence of tantrums. While children with this syndrome display a visually observed overgrowth, they often make an impression of older and more developed children than they actually are. Therefore, environmentally generated frustration for a child is believed to be the principal cause of the occurring behavioral problems [13]. 

If the syndrome-specific behavioral features are preferred to be assessed, a representative sample and a control group are essential. Most importantly, both factors should be matched by similar intellectual level, age, and gender in order to obtain an exact behavioral profile. For instance, in 2015, Sheth et al. analyzed behavioral questionnaires from 38 children and adults with Sotos syndrome and compared them to cohorts with Down syndrome, Prader–Willi syndrome (PWS), and autism spectrum disorder (ASD). Interestingly, a combination of self-injuring, stereotyped, and destructive behaviors occurred in over 40% of individuals with Sotos syndrome, higher than in those with Down syndrome. The self-injurious and destructive behaviors were similar to the ASD and PWS groups, but stereotyped behaviors were less common in the Sotos group. Impulsivity and hyperactivity were most frequently observed in Sotos participants [16]. Thus, progression in comparative methodology in assessing aggression and loss of self-control could lead to advancing the neurobehavioral phenotype of Sotos syndrome.

### 3.5. Autistic Features

Impairment in intellect and communication abilities are believed to play a solid part in the behavioral development of children with Sotos syndrome. The behavioral phenotype comprises the manifestation of autistic features that affect two domains: social communication/interaction and restrictive, repetitive patterns of behavior [24].

Lane C. et al. published an analysis in 2019 in which the relationship between language and social level was investigated. Despite the results referring to parents’ observations, Sotos children demonstrated notable difficulties in applying pragmatic and non-verbal language. When comparing children with Sotos and children with Williams syndrome, struggles in forming and understanding social relations were reported. These findings linked Sotos syndrome to the autism spectrum disorder (ASD) profile [17]. Another earlier study by Lane et al. in 2016 mentioned that, according to reviewed cases, ASD could be a pervasive feature of Sotos syndrome [13]. However, the severity of ASD was not compared within the intellectual disabilities population and the studies lacked systematicity. 

Findings by Sheth et al. in 2015 highlighted that the majority (∼70%) of Sotos patients meet the cut-off level for ASD based on the Social Communication Questionnaire [16]. A similar specific evaluation of ASD in Sotos syndrome was recently conducted in Lane et al.’s cohort study in 2017. The results demonstrated that the symptomatology of ASD is exceedingly common in the majority (83%) of the Sotos population. ASD intensity differed when comparing younger (2.5–5 years) with older children (5–19 years) and adults (20+ years), but there were no gender-specific symptoms. Children in early childhood displayed a decreased severity of ASD, as well as adults, whereas ASD severity was increased for adolescents. Additionally, genetic variants in the *NSD1* gene were implied to contribute to ASD manifestation in Sotos patients [18]. 

As social engagement is deeply encouraged in managing autistic features, Siracusano M. et al. in 2022 investigated children with Sotos syndrome and ASD during the COVID-19 pandemic when social contact was restricted. The short-term results indicated that the social skills (cognition and communication skills) of Sotos children were notably impaired as a result of social distancing [19]. This indicated that improving social responsiveness for children with Sotos could help in managing the autistic phenotype.

### 3.6. Attention Deficit Hyperactivity Disorder (ADHD)

One more discussed neurobehavioral feature of Sotos syndrome is attention deficit and hyperactivity disorder (ADHD). ADHD in children is usually suspected when signs of inattention, hyperactivity, impulsivity, oppositionality, or poor academic progress appear [25]. 

In Lane et al.’s study in 2016, ADHD symptoms were noted for some children with Sotos during the behavioral assessment. Individuals, according to parental reports, could not maintain focus on given tasks, were overactive, and demonstrated a lack of inhibition, thus resulting in clinical ADHD diagnosis [13]. Unfortunately, both Lane’s research and the reviewed literature in her study lacked greater sample sizes and proper control groups even though findings may relate ADHD to Sotos syndrome.

### 3.7. Anxiety

Anxiety disorder, the most common mental health disorder in children, together with ASD, construct a predominant behavioral profile for Sotos individuals. In 2003, Sarimski K. et al. analyzed the response of Sotos children with learning disabilities in school and social gatherings. The unusual social environment provoked more separation anxiety for Sotos children; they felt more anxious in dealing with new situations when compared with a matched control group by age and IQ [15].

Later, in 2016, Lane C. et al. reviewed earlier studies conducted by K. Sarimski, Rutter and Cole and mentioned phobias provoked by anxiety in Sotos individuals [13]. Nevertheless the highlighted anxiety in the Sotos population needed more evidence to be thoroughly confirmed.

Another thesis published by Goulding-Talbot J. in 2022 referred to elevated rates of anxiety in Sotos patients and linked this feature with ASD [20]. Based on knowledge about other genetic syndromes associated with ASD, Sotos syndrome would not be an exception when it comes to higher anxiety rates. However, the specifics of anxiety may still differ between neurodevelopmental genetic syndromes.

### 3.8. Unusual Sleep Habits

Sleep habits of children with Sotos syndrome are briefly discussed in earlier studies even though they are inseparable from behavioral changes, such as ADHD, aggression/tantrums, or anxiety.

Mouridsen S.E. et al. in 2002 mentioned the occurring sleep issues, typically awakening early in the morning, for about 69% of the researched Sotos children [11]. But only the recent cross-sectional study by Stafford C.E. et al. in 2021 aimed to characterize sleep habits for these patients in detail. The findings implied that Sotos children’s sleep was more disturbed, and their sleeping patterns differed from the ones of children with other intellectual disabilities. In addition, as proposed in earlier studies, Sotos children had early bed and rise times, routinely used transitional objects, and showed repetitive motions at sleep onset. Interestingly, sleep duration did not decrease with age nor did Sotos children experience sleepwalking or night terrors. Individuals predominantly fell asleep within 20 min at the same time each night in their own bed [21].

## 4. Discussion

This review provides different aspects of Sotos syndrome, comprising genetic, developmental, neuropsychiatric, and psychosocial perspectives. Sotos syndrome, defined as a neurodevelopmental genetic disorder, presents various neuropsychiatric symptoms [13]. Children’s intellectual capacity demonstrates a delay ranging from mild to severe IQ scores and verbal abilities being stronger than non-verbal abilities [12,13,22]. Further research on speech and language development is needed. Speech and language delays in early childhood are not as significant as they seemed in earlier studies that analyzed parental reports [13,15]. However, receptive language is considered to be more dominant than expressive language since this feature correlates with verbal IQ. It is important to assist children with Sotos syndrome in developing structural language skills, including syntax, semantics, pronunciation, grammar, and the production of coherent speech [17]. The involvement of speech and language therapists into multidisciplinary teams is therefore important. 

The behavioral profile of Sotos includes a decreased barrier in tolerating anger-provoking behavior, higher possible rates of ADHD, and anxiety [13]. The majority of people with Sotos syndrome show signs of autism [19,24]. Autism spectrum disorder depends on the individual’s age, with the most prominent being observed during adolescence, and manifests in a lack of socializing and adaptivity to new environments. Genetic correlation with ASD is also important in predicting the autism severity of Sotos patients [13]. Establishing systematic support for family members is still little explored in the literature. How to manage problems related to anger and inappropriate behavior of patients with Sotos syndrome is a challenge for medical service organizers and providers, and there is a lack of good practice examples in the literature. Specialists such as neonatologists, pediatricians, family physicians, child and adolescent psychiatrists, and others who may encounter individuals with Sotos syndrome should be able to suspect and recognize genetic syndromes. Common signs that can alert doctors to genetic diseases include: dysmorphic features, multiple anomalies in one patient, neurocognitive impairment, and a family history that is suggestive of a hereditary disease [26]. In the relatively sparse literature on Sotos syndrome, there is clarity about the genetic causes and clinical manifestations. 

Descriptions of clinical cases of Sotos syndrome provide insight into how it manifests in young or older patients and various difficulties the patients and their families face. A case report by Gomes-Silva et al. (2016) documented a child diagnosed with Sotos syndrome and described the primary clinical features, disease-specific craniofacial, oral, and dental findings, and dental care management of this patient [27]. In a case report of a male Saudi patient who presented with abnormal rapid growth, delayed motor and mental milestones, aggressive behavior, obsession to close doors, nail biting, defective attention, and hyperactivity, a novel heterozygous deletion of all exons 1 to 23 of the NSD1 gene was detected, emphasizing that besides its characteristic clinical picture, molecular genetic testing is also recommended [28]. A recent report from China described the case of a 4-year-old female child with Sotos syndrome caused by an NSD1 gene nonsense mutation who showed typical facial features, hand deformities, and seizure [29]. A case report of a 47-year-old female with Sotos syndrome and focal-onset seizures in the left temporal lobe demonstrated that resective surgery may play a significant role in improving patient quality of life and seizure control [30]. 

Another case report of Sotos syndrome discussed a rare but important pitfall in the presurgical work-up of temporal lobe epilepsy, describing the critical value of thorough presurgical diagnostics, including genetic testing, in apparently straightforward cases of lesional epilepsy to rule out an underlying genetic etiology that may not be treated by surgery [31]. The phenotype in adults with Sotos syndrome is not yet well described; associated clinical features include scoliosis, seizures, renal anomalies, and cardiac anomalies [9]. Although all Sotos syndrome patients should be monitored for scoliosis, recent research emphasized that patients with NSD1 microdeletions have a higher probability of scoliosis development and progression, which may require early intervention.

The treatment approach for Sotos syndrome involves coordination among multiple medical specialties and the syndrome is mostly symptomatic. In the neonatal period, management of Sotos syndrome often involves phototherapy for jaundice, treatment of feeding difficulties, gastroesophageal reflux, and hypoglycemia. Throughout infancy and childhood, regular pediatric follow-ups are essential to address clinical events like constipation, respiratory infections, scoliosis, and seizures, as well as the potential risk of tumors [8]. Early intervention programs encompassing various therapies are vital for developmental support, with ongoing psychological and psychiatric support required as the child progresses through school and into adulthood to address any emerging challenges. Families need guided help to accept the diagnosis and long-term implementation of medical recommendations. Patients with Sotos syndrome and their families need complex and systematic management and care. These aspects of the multidisciplinary approach are still little researched. 

## 5. Conclusions

Therefore, further research on the connection between cognition, behavior, emotions, and molecular changes is necessary to establish a complete neuropsychiatric profile of Sotos syndrome. Good practice examples in the organization of relevant complex help could be more broadly described and investigated. A multidisciplinary team comprising geneticists, pediatricians, neurologists, and speech, rehabilitation, and mental health specialists is needed for the organization of complex systematic help during the developmental trajectory of children with Sotos syndrome. Still, there is a lack of research on the developmental characteristics of these children and the possibilities of improving psychosocial adaptation by providing multidisciplinary long-term medical, educational, and social care.

## Figures and Tables

**Table 1:** Selected articles on neuropsychiatric aspects of Sotos syndrome.

Study	Author, Year	Section in Which the Article is Included	Type of the Study	Number of Patients with Sotos Syndrome Included
Disrupted epigenetics in the Sotos syndrome neurobehavioral phenotype	Harris, J.R. et al., 2019 [12]	Neurobehavioral phenotype	Literature review	-
Cognition and Behaviour in Sotos Syndrome: A Systematic Review	Lane, C. et al., 2016 [13]	Intelligence quotient, language, aggression and tantrums, autistic features, attention deficit hyperactivity disorder, anxiety	Systematic review	247
The cognitive profile of Sotos syndrome	Lane, C. et al., 2019 [14]	Intelligence quotient	Cross-sectional	52
Behavioural and emotional characteristics in children with Sotos syndrome and learning disabilities	Sarimski, K., 2003 [15]	Language, anxiety	Cross-sectional	27
The behavioral characteristics of Sotos syndrome	Sheth K. et al., 2015 [16]	Language, aggression and tantrums, autistic features	Cross-sectional	38
Parent-Reported Communication Abilities of Children with Sotos Syndrome: Evidence from the Children’s Communication Checklist-2	Lane, C. et al., 2019 [17]	Autistic features	Cross-sectional	31
Characteristics of Autism Spectrum Disorder in Sotos Syndrome	Lane, C. et al., 2017 [18]	Autistic features	Cross-sectional	78
COVID-19 and social responsiveness: A comparison between children with Sotos syndrome and autism	Siracusano, M. et al., 2021 [19]	Autistic features	Cross-sectional	12
An exploration of the psychological and behavioural profile of specific genetic syndromes, including Malan Syndrome and Sotos Syndrome	Goulding-Talbot, J. 2022 [20]	Anxiety	Cross-sectional	9
Characterization of sleep habits of children with Sotos syndrome	Stafford, C.F., et al., 2021 [21]	Unusual sleep habits	Cross-sectional	49
Neuropsychiatric aspects of Sotos syndrome. A review and two case illustration	Mouridsen, S.E. et al., 2002 [11]	Unusual sleep habits	Literature review and case reports	-

## Data Availability

The data presented in this study are available upon request from the corresponding author. The data are not publicly available due to privacy.
